# *Lactobacillus gasseri* SBT2055 inhibits adipose tissue inflammation and intestinal permeability in mice fed a high-fat diet

**DOI:** 10.1017/jns.2016.12

**Published:** 2016-05-30

**Authors:** Michio Kawano, Masaya Miyoshi, Akihiro Ogawa, Fumihiko Sakai, Yukio Kadooka

**Affiliations:** Milk Science Research Institute, Megmilk Snow Brand Co. Ltd, 1-1-2 Minamidai, Kawagoe, Saitama, Japan

**Keywords:** *Lactobacillus gasseri* SBT2055, Intestinal barrier function, Anti-obesity effects, Anti-inflammation effects, Diet-induced obesity, FBS, fetal bovine serum, FCM, flow cytometry buffer, FD-4, fluorescein isothiocyanate–dextran, FITC, fluorescein isothiocyanate, HFD, high-fat diet, HFD-LG, high-fat diet containing *Lactobacillus gasseri* SBT2055, IFN-γ, interferon-γ, LPS, lipopolysaccharide, LY, Lucifer yellow, M1, classically activated macrophages, M2, alternatively activated macrophages, NFD, normal-fat diet, SVF, stromal–vascular fraction, TEER, trans-epithelial electrical resistance

## Abstract

The probiotic *Lactobacillus gasseri* SBT2055 (LG2055) has anti-obesity effects. Obesity is closely correlated with inflammation in adipose tissue, and maintaining adipose tissue in a less-inflamed state requires intestinal integrity or a barrier function to protect the intestine from the disruption that can be caused by a high-fat diet (HFD). Here, we examined the anti-inflammatory and intestinal barrier-protecting effects of LG2055 in C57BL/6 mice fed a normal-fat diet (NFD), HFD, or the HFD containing LG2055 (HFD-LG) for 21 weeks. HFD-LG intake significantly prevented HFD-induced increases in body weight, visceral fat mass, and the ratio of inflammatory-type macrophages to anti-inflammatory ones in adipose tissue. Mice fed the HFD showed higher intestinal permeability to a fluorescent dextran administered by oral administration and an elevated concentration of antibodies specific to lipopolysaccharides (LPS) in the blood compared with those fed the NFD, suggesting an increased penetration of the gut contents into the systemic circulation. These elevations of intestinal permeability and anti-LPS antibody levels were significantly suppressed in mice fed the HFD-LG. Moreover, treatment with LG2055 cells suppressed an increase in the cytokine-induced permeability of Caco-2 cell monolayers. These results suggest that LG2055 improves the intestinal integrity, reducing the entry of inflammatory substances like LPS from the intestine, which may lead to decreased inflammation in adipose tissue.

Obesity is often associated with chronic low-grade inflammation that is a cause of insulin resistance that may in turn cause metabolic disorders^(^^1^^)^, in which pro-inflammatory cytokines, such as TNF-α and IL-6, inhibit insulin signalling in the muscle, liver and other organs^(^^2^^)^. In addition to pro-inflammatory cytokine expression, infiltration of macrophages and lymphocytes into adipose tissue is also critical for low-grade inflammation^(^^2^^)^.

Mechanisms by which obesity-associated low-grade inflammation is initiated in adipose tissue remain controversial. Recent studies showed that lipopolysaccharides (LPS) derived from Gram-negative bacteria in the gut could be a trigger for adipose tissue inflammation and insulin resistance in obesity^(^^3^^,^^4^^)^. In a healthy intestine, the translocation of LPS from the gut lumen into the circulation is prevented by the intestinal epithelial barrier function. However, once the intestinal barrier is disrupted under conditions such as alcohol intake^(^^5^^)^, dextran sodium sulfate treatment^(^^6^^)^, or restraint stress^(^^7^^)^, LPS levels in the blood increase. Cani *et al*. reported that a high-fat diet or an obese state disrupted the intestinal barrier and increased LPS levels in the blood^(^^4^^)^. Furthermore, they demonstrated that mice administered LPS exhibited fat pad weight gain, inflammation in visceral adipose tissue and insulin resistance, all of which are common in obesity^(^^3^^)^. In human studies, the plasma LPS concentration is positively correlated with visceral fat volume and indexes of insulin resistance (for example, homeostatic model assessment of insulin resistance (HOMA-IR) index and HbA1c)^(^^8^^,^^9^^)^. Therefore, it is conceivable that the intestinal barrier disruption caused by HFD intake or obesity induces the entry of LPS into the circulation, leading to adipose tissue inflammation and insulin resistance.

*Lactobacillus gasseri* SBT2055 (LG2055) is a probiotic bacterium of human origin that has bile acid tolerance and can colonise the intestine and improve the intestinal environment^(^^10^^–^^12^^)^. In addition, LG2055 has anti-obesity effects; for example, LG2055-containing fermented milk and LG2055 cells themselves prevented the enlargement of adipocytes and an increase in abdominal fat volume in rats, mice and humans^(^^13^^–^^16^^)^. Furthermore, LG2055 improved the inflammatory status of the body and adipose tissue: fermented milk containing LG2055 prevented the elevation of the blood level of soluble intercellular adhesion molecule-1 (sICAM-1), a circulatory inflammatory marker, in rats fed a 10 % fat diet^(^^17^^)^. LG2055 cells also prevented an increase in the mRNA expression of chemokine CC motif ligand 2 (CCL2) in adipose tissue, which contributes to macrophage infiltration into the tissue, in mice fed a 10 % fat diet^(^^15^^)^. These results suggest that LG2055 has the ability to ameliorate systemic and adipose tissue inflammation in obesity. However, it has not been determined if LG2055 prevents the intestinal barrier disruption that contributes to the development of adipose tissue inflammation in obesity.

In the present study, we examined the anti-inflammatory and intestinal barrier-protecting effects of LG2055 in mice fed a high-fat (20 % (w/w) fat) diet containing LG2055 cells. We also used an intestinal epithelial cell culture model whose permeability was enhanced by inflammatory cytokines to determine whether LG2055 prevents the permeability.

## Experimental methods

### Preparation of LG2055 cells

LG2055 is a bacterial strain deposited in the International Patent Organism Depository, National Institute of Advanced Industrial Science and Technology (Tsukuba, Japan). LG2055 was cultured in a sterile de Man, Rogosa and Sharpe (MRS) broth (BD Bioscience) at 37°C for 16 h. After cultivation, bacterial cells were collected by centrifugation at 4620 ***g*** for 10 min at 4°C, and washed twice with saline and once with sterile water. To prepare the experimental diet for the mice, LG2055 cells were suspended in a 10 % (w/w) lactose solution to reduce the loss of cell viability before lyophilisation. The lyophilised powder was weighed and the net LG2055 weight was calculated by subtracting the weight of lactose in the original lactose solution from the total weight. Additional lactose was then added to the lyophilised powder so that it contained equal weights of LG2055 and lactose. The LG2055 powder thus prepared contained 1·4 × 10^11^ colony-forming units (cfu) of LG2055/g of powder, and was stored at −30°C. For the cell culture assay, washed LG2055 cells were lyophilised without lactose and stored at −80°C until use.

### Mice and diets

Male C57BL/6J mice, aged 7 weeks, purchased from Charles River Japan Inc., were housed individually in plastic cages in an air-conditioned specific pathogen-free room (21–24°C, lights on 07.00–19.00 hours) with free access to food and deionised water. The mice were acclimatised to a powder diet for 1 week, then divided into three groups (sixteen mice per group): those fed a normal-fat diet (NFD), a high-fat diet (HFD), or a high-fat diet containing LG2055 (HFD-LG).

Experimental diets were prepared according to the American Institute of Nutrition (AIN)-76 formula with some modifications^(^^15^^,^^18^^)^. Compositions of the experimental diets are shown in Table 1. These diets contained 20 % skimmed milk, which is not used in the original AIN-76 formula. The protein and carbohydrate contents that were increased by the addition of skimmed milk were balanced by decreasing the amounts of casein and sucrose, respectively. The NFD contained 5 % maize oil (11·7 % energy from fat) as commonly described, and the HFD and HFD-LG contained 2 % maize oil and 18 % lard (39·2 % energy from fat). The weight difference in the fat content among the diets was compensated for with sucrose. The HFD-LG contained 1 % of the LG2055 powder to provide 1·4 × 10^9^ cfu of viable LG2055 cells with 0·5 % lactose per 1 g of the diet. The NFD and HFD were supplemented with 0·5 % lactose to equalise the lactose content among the diets. Mice were fed these diets and their body weight was monitored once a week. After 18 weeks of feeding, an intestinal permeability assay as described later was performed. At the end of the experimental period of 21 weeks, mice were fasted for 12 h, anaesthetised with isoflurane, and exsanguinated via the portal vein. The serum prepared from the portal blood was stored at −80°C until use. The liver and the mesenteric, perirenal/retroperitoneal and epididymal fat were removed from each mouse and weighed.

**Table 1.** Diet composition

	(%, w/w)		
Ingredient	NFD*	HFD†	HFD-LG‡
Skimmed milk	20·0	20·0	20·0
Casein	12·5	12·5	12·5
dl-Methionine	0·3	0·3	0·3
α-Maize starch	15·0	15·0	15·0
Sucrose	37·2	22·2	21·7
Maize oil	5·0	2·0	2·0
Lard	–	18·0	18·0
Cellulose	5·0	5·0	5·0
AIN-76 mineral mixture	3·5	3·5	3·5
AIN-76 vitamin mixture§	1·0	1·0	1·0
*Lactbacillus gasseri* SBT2055	–	–	0·5
Lactose ||	0·5	0·5	0·5

NFD, normal-fat diet; HFD, high-fat diet; HFD-LG, high-fat diet containing LG2055; AIN, American Institute of Nutrition.

* 5 % (w/w) fat, 11·7 % energy from fat.

† 20 % (w/w) fat, 39·2 % energy from fat.

‡ HFD containing *Lactobacillus gasseri* SBT2055 (1·4 × 10^9^ colony-forming units/g).

§ AIN-76 vitamin mixture containing choline bitartrate.

|| Lactose was used in the lyophilisation processes of LG2055 cells to reduce viability loss.

The protocol was submitted to, and approved by, the Animal Care and Use Committee of the Milk Science Research Institute of Megmilk Snow Brand Co., Ltd, whose guidelines are based on those of the Science Council of Japan.

### Isolation of stromal–vascular fraction from epididymal fat

Isolation of the stromal–vascular fraction (SVF) was performed as previously described^(^^19^^)^. Briefly, epididymal adipose tissues from mice were dissected and suspended in an ice-cold Roswell Park Memorial Institute (RPMI) medium (Gibco) supplemented with fetal bovine serum (FBS) and antibiotics, then transferred into PBS supplemented with 1 µg/ml heparin. Adipose tissues were then cut into fine pieces that were centrifuged. Floating pieces of adipose tissue were digested at 37°C for 20 min with 1 mg/ml collagenase (Sigma) and 10 µg/ml DNase I (Roche) in RPMI medium. The samples were passed through a 70 µm mesh and centrifuged at 620 ***g*** for 3 min. Pellets thus collected were re-suspended into an erythrocyte lysis buffer (168 mm-NH_4_Cl, 10 mm-KHCO_3_ and 0·08 mm-EDTA), incubated for 3 min, and washed with an ice-cold flow cytometry buffer (FCM buffer; 1 % FBS, 0·05 % sodium azide in PBS) to obtain SVF.

### Flow cytometry analysis

The SVF (1 × 10^5−6^ cells per assay tube) was washed with PBS and centrifuged at 620 ***g*** for 3 min. Pellets were suspended with Zombie Aqua (BioLegend) live–dead discriminating dye, diluted with PBS, and incubated at room temperature for 15 min in the dark. Cells were washed with FCM buffer and fixed with 0·1 % paraformaldehyde/PBS at room temperature for 20 min in the dark. Fixed cells were washed with FCM buffer, centrifuged and suspended in an anti-mouse CD16/32 (eBioscience)/FCM buffer, followed by incubation at 4°C for 10 min in the dark. Cells were washed with FCM buffer then stained with antibodies interacting with cell-surface antigens or matching control isotypes for 30 min. The following antibodies were used: anti-mouse fluorescein isothiocyanate (FITC)-labelled CD11b (BD Bioscience), allophycocyanin-labelled CD11c (BD Bioscience), phycoerythrin-labelled CD206 (BD Bioscience), Brilliant Violet 421-labelled F4/80 (BioLegend), Pacific Blue-labelled CD4 (BD Bioscience) or FITC-labelled CD8a (eBioscience). The cells were rinsed and re-suspended in FCM buffer and analysed using a FACS Cant II flow cytometer (BD Bioscience). A cell population with CD11b-, F4/80- and CD11c-positive but CD206-negative was defined as a pro-inflammatory macrophage population (classically activated macrophages or M1 subtype), and that with CD11b-, F4/80- and CD206-positive but CD11c-negative was defined as an anti-inflammatory macrophage population (alternatively activated macrophages or M2 subtype). CD4-positive but CD8-negative T cells were defined as CD4-positive T cell subpopulation and CD8-positive but CD4-negative T cells were defined as CD8-positive T cell subpopulation.

### *In vivo* intestinal permeability assay

Intestinal permeability was measured as previously reported^(^^20^^)^ with some modifications. Mice fed experimental diets for 18 weeks were fasted for 12 h and orally given FITC–dextran with average molecular weight of 3000–5000 Da (FD-4) (Sigma) in saline (500 mg/kg body weight, 125 mg/ml). After 1 and 4 h, 100 µl of blood were collected from the orbital sinus. Plasma was obtained by the addition of 10 µg/ml heparin and centrifugation at 1200 ***g*** for 30 min at 4°C. The plasma was diluted 3-fold with PBS and analysed for FD-4 concentration with a fluorescence spectrophotometer (Varioskan Flash; Thermo Scientific) at an excitation wavelength of 485 nm and an emission wavelength of 535 nm.

### Serum anti-lipopolysaccharide antibody assay

Serum obtained from portal blood was analysed for anti-LPS IgG levels using a commercial ELISA kit from Chondrex.

### Cell culture

Caco-2 cells were purchased from the American Type Culture Collection (ATCC). Dulbecco's modified Eagle's medium (DMEM) (Sigma) supplemented with 10 % FBS, 1 % non-essential amino acids and antibiotics was used as a growth medium. The cells were seeded into a twenty-four-well cell culture insert (BD Bioscience) (5 × 10^4^ cells/cm^2^) that was pre-coated with collagen (Nitta Gelatin). After 21 d, cells were differentiated into an epithelial cell-like phenotype and used for the following assay.

### Paracellular permeability assay using Caco-2 cell monolayers

Paracellular permeability was determined by trans-epithelial electrical resistance (TEER) and the fluorescent dye flux across Caco-2 cell monolayers. TEER was measured using Millicell ERS-2 (Millipore) and expressed in Ω × cm^2^. The fluorescent dye flux was measured using an impermeable fluorescent Lucifer yellow dye (LY; Invitrogen). LY (100 µm) was added to the apical side of Caco-2 monolayers, and the concentration of LY infiltrated into the basal side of Caco-2 monolayers was measured after 3 h using a fluorescence spectrophotometer (Varioskan Flash; Thermo Scientific) at an excitation wavelength of 428 nm and an emission wavelength of 535 nm. The LY flux was expressed in pmol/h × cm^2^.

LG2055 cells (1 mg/ml) suspended in DMEM without FBS were added to the apical side of Caco-2 monolayers and interferon-γ (IFN-γ, 50 ng/ml; Pepro Tech) and TNF-α (10 ng/ml, Pepro Tech) were simultaneously added to the basal side for 3 d to promote paracellular permeability. TEER was monitored daily and the LY flux was measured 72 h after treatment with LG2055 and the cytokines.

### Statistical analysis

Data were expressed as mean values and standard deviations. Statistical analyses were performed by using PASW Statistics 17.0 (SPSS, Inc.) and StatView software package 5.0 (SAS Institute, Inc.). Data were analysed by one-way ANOVA, followed by Dunnett's test, which compared the NFD group or the HFD-LG group with the HFD group in *in vivo* experiments, and the control group or the IFN-γ + TNF-α + LG2055 group with the IFN-γ + TNF-α group in *in vitro* experiments. *P* values < 0·05 were considered statistically significant. Correlation analysis was assessed by the Pearson's test using StatView software.

## Results

### LG2055 prevents high-fat diet-induced weight gain of the body and adipose tissues

The quantity of the given diet consumed and the weights of the total body, adipose tissues and liver are shown in Table 2. Despite a smaller intake quantity in the HFD and HFD-LG groups than in the NFD group, energy intake was greater in the HFD and HFD-LG groups than in the NFD group, with no significant differences in either diet or energy intake between the HFD and HFD-LG groups. The HFD group exhibited significantly greater weights of the body, adipose tissues and liver than the NFD group. In a comparison between the HFD and HFD-LG groups, the HFD-LG group showed significantly lower weights of the body, mesenteric and perirenal/retroperitoneal adipose tissues than the HFD group. Thus, LG2055 prevented HFD-induced increases in the body and adipose tissue weights, despite the same energy intake by the HFD and HFD-LG groups.

**Table 2.** Diet intake, body weight, adipose tissue weight and liver weight† (Mean values and standard deviations; sixteen mice per group)

	Experimental groups					
	NFD		HFD		HFD-LG	
Parameters	Mean	sd	Mean	sd	Mean	sd
Average diet intake (g/d)	3·06*	0·07	2·86	0·22	2·87	0·17
Average energy intake (kJ/d)	49·17*	1·12	54·96	4·17	55·11	3·20
Initial body weight (g)	23·91	1·13	23·92	1·62	23·93	1·30
Final body weight (g)	32·61*	1·94	42·04	4·67	39·08*	2·11
Adipose tissue weight (g)						
Mesenteric	0·51*	0·10	1·00	0·38	0·79*	0·16
Epididymal	1·24*	0·40	2·57	0·47	2·38	0·51
Perirenal/retroperitoneal	0·53*	0·21	1·29	0·30	1·07*	0·24
Total	2·26*	0·73	4·87	0·94	4·24	0·85
Liver weight (g)	1·01*	0·06	1·13	0·23	1·03	0·12

NFD, normal-fat diet; HFD, high-fat diet; HFD-LG, high-fat diet containing LG2055.

* Mean value was significantly different from that of the HFD group (*P* < 0·05).

† Data were analysed by one-way ANOVA, followed by Dunnett's multiple-comparison test. All parameters except ‘Initial body weight’ were determined 21 weeks after the start of feeding the experimental diets.

### LG2055 prevents high-fat diet-augmented inflammatory status in epididymal adipose tissue

**Fig. 1.**
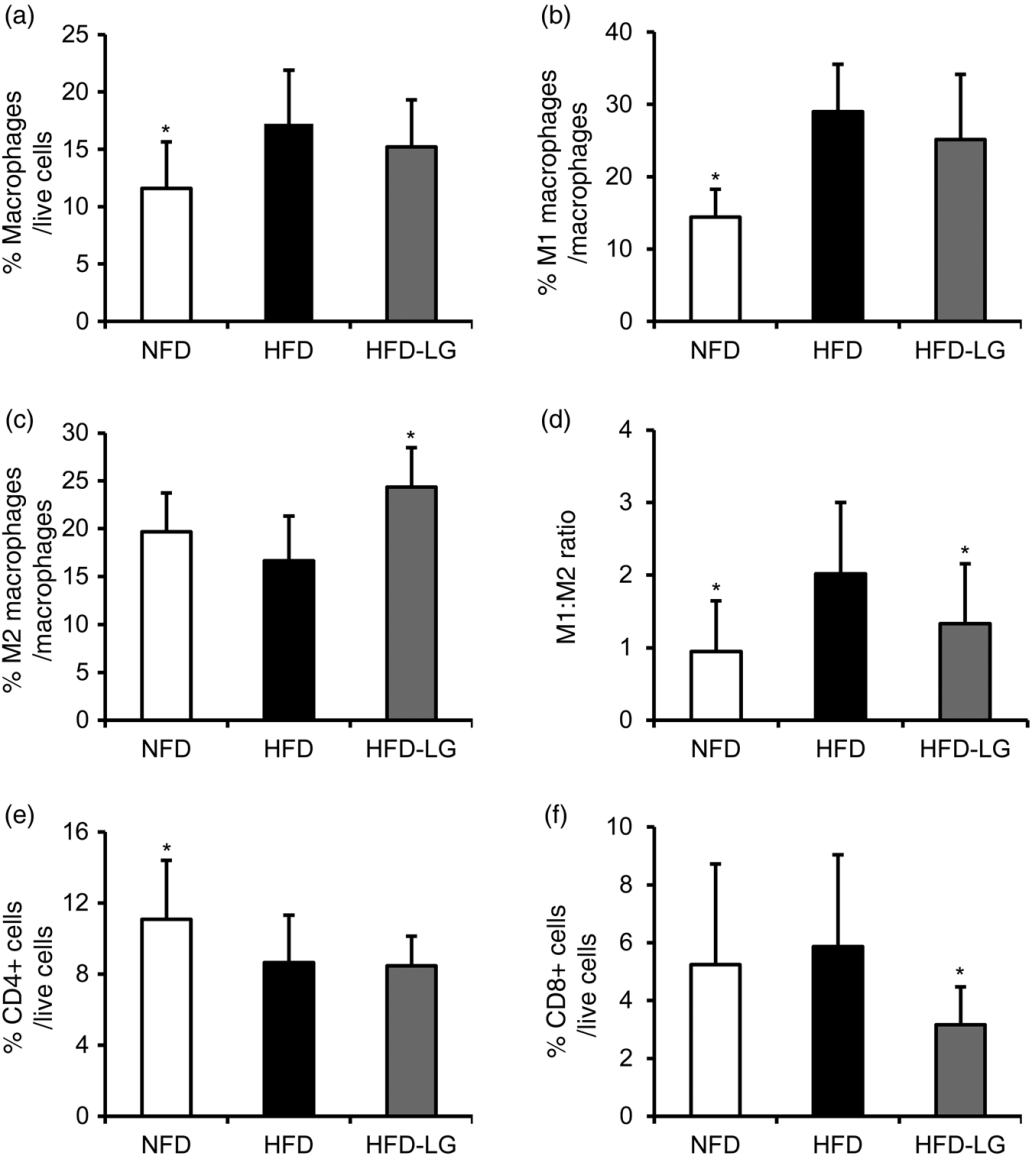
Effect of *Lactobacillus gasseri* SBT2055 (LG2055) on immune cell populations in epididymal adipose tissue. The stromal–vascular fraction (SVF) was prepared from epididymal fat tissue of mice fed a normal-fat diet (NFD), high-fat diet (HFD) or the HFD containing LG2055 (HFD-LG) for 21 weeks, and cells in the SVF were analysed by flow cytometry. (a) Percentage of F4/80- and CD11b-positive cells defined as macrophages in live cells of the SVF. Percentages of CD11c-positive and CD206-negative macrophages defined as classically activated (M1) macrophages (b), and CD11c-negative and CD206-positive macrophages defined as alternatively activated (M2) macrophages (c) in total macrophages. (d) Ratio of M1 macrophages to M2 macrophages. (e) Percentage of CD4-positive cells in live cells of the SVF. (f) Percentage of CD8-positive cells in live cells of the SVF. Values are means, with standard deviations represented by vertical bars (*n* 15–16 in each group). Data were analysed by one-way ANOVA, followed by Dunnett's multiple-comparison test. * Mean value was significantly different from that of the HFD group (*P* < 0·05).

The immune cell population in epididymal adipose tissue was analysed. There were no effects of LG2055 on the total number of stromal–vascular live cells (data not shown). The percentage of adipose tissue macrophages in stromal–vascular live cells was increased in the HFD group compared with the NFD group (Fig. 1(a)). The percentage of classically activated M1 macrophages, which have pro-inflammatory properties, was increased in the HFD group (Fig. 1(b)), while that of alternatively activated M2 macrophages, which have anti-inflammatory properties, was not affected by HFD (Fig. 1(c)). The M1:M2 ratio, which represents the degree of inflammatory status in adipose tissue, was increased by the HFD (Fig. 1(d)). Compared with the HFD group, the HFD-LG group showed a significantly larger proportion of anti-inflammatory M2 macrophages (Fig. 1(c)) and a significantly lower M1:M2 ratio (Fig. 1(d)), suggesting that the intake of the HFD-LG ameliorates an HFD-induced disposition in macrophage composition to a pro-inflammatory status.

We also analysed the T cell subpopulation, which is involved in inflammation in adipose tissue. The HFD reduced the CD4-positive T cell subpopulation in adipose tissue compared with the NFD (Fig. 1(e)), while the CD8-positive T cells (Fig. 1(f)) did not significantly differ between the HFD and NFD groups. Compared with the HFD group, the HFD-LG group had a decreased proportion of CD8-positive cells (Fig. 1(f)), suggesting that LG2055 modulated part of the T cell subpopulation in the adipose tissue.

Taken together, these results suggest that LG2055 could prevent the inflammation in the adipose tissue caused by HFD.

### LG2055 prevents high-fat diet-induced intestinal permeability

**Fig. 2.**
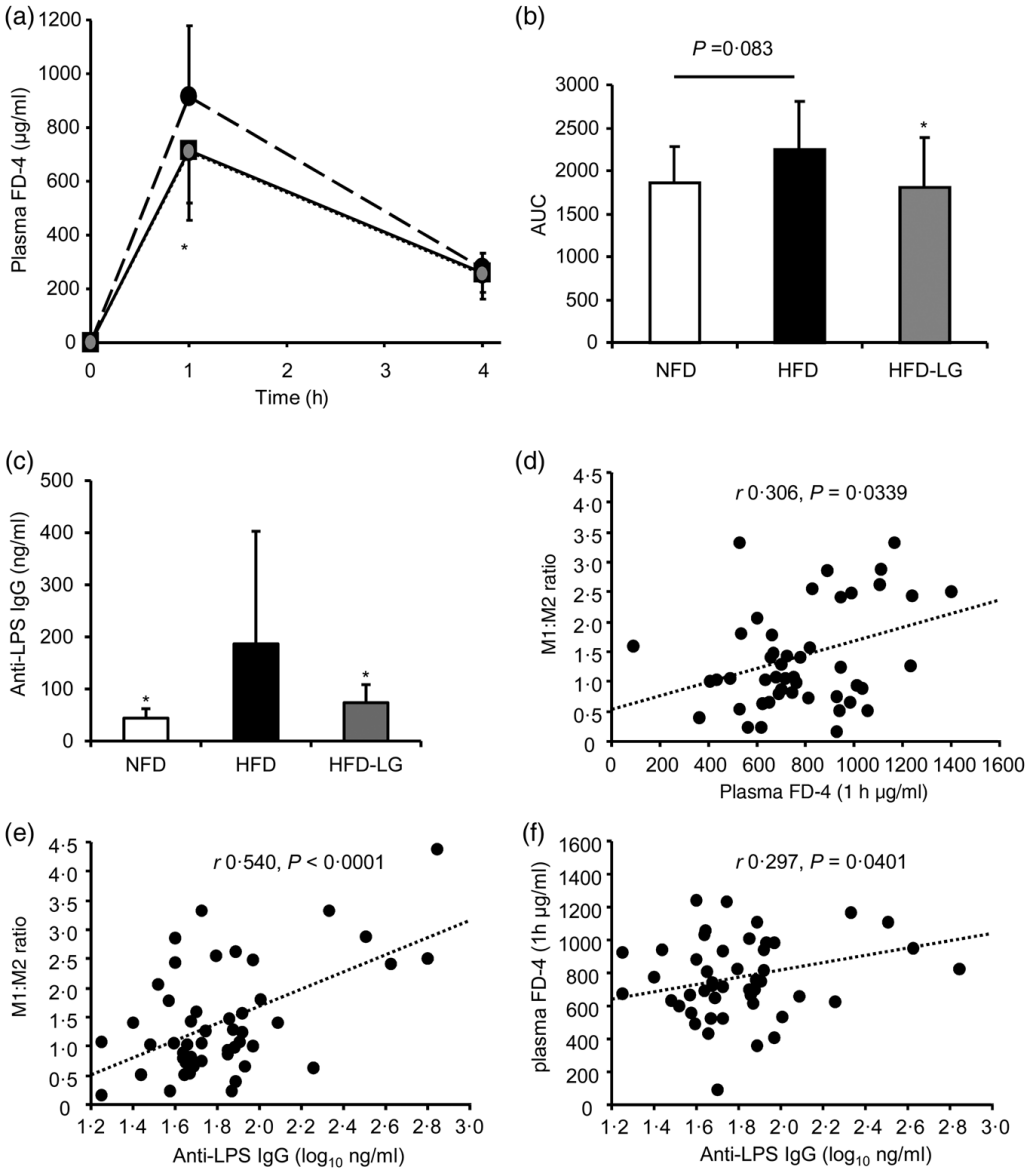
Effect of *Lactobacillus gasseri* SBT2055 (LG2055) on intestinal permeability and anti-lipopolysaccharide (LPS) antibody response. (a) Plasma fluorescein isothiocyanate–dextran (FD-4) levels were measured 1 and 4 h after oral administration of FD-4 in mice fed a normal-fat diet (NFD; –□–), a high-fat diet (HFD; –●–) or the HFD containing LG2055 (HFD-LG; --●◌--) for 18 weeks. (b) AUC of plasma FD-4 levels in each group. (c) Anti-LPS IgG levels in the serum obtained from the portal vein. Values are means, with standard deviations represented by vertical bars (*n* 16 in each group). Data were analysed by one-way ANOVA, followed by Dunnett's multiple-comparison test. * Mean value was significantly different from that of the HFD group (*P* < 0·05). Correlations between the plasma FD-4, anti-LPS antibody and adipose M1:M2 ratio parameters were plotted (d–f) with Pearson's *r* correlation and the corresponding *P* value.

We analysed the intestinal permeability to FD-4, a fluorescein-labelled dextran, in mice fed the experimental diets for 18 weeks. The HFD group showed a higher plasma concentration of FD-4 at 1 h after oral administration of FD-4 than the NFD group, indicating disruption of the intestinal barrier by the HFD (Fig. 2(a)). In contrast, at the same time point, the plasma FD-4 concentration in the HFD-LG group was lower than that in the HFD group, and the concentration was approximately equal to that in the NFD group (Fig. 2(a)). The FD-4 area under the concentration-time curve (AUC 0–4 h) also showed a significantly lower value in the HFD-LG group compared with the HFD group (Fig. 2(b)). Thus, LG2055 was shown to prevent the increase in the intestinal permeability caused by the HFD.

To examine whether the protective effect of LG2055 on the intestinal barrier affects the entry of bacterial LPS into the portal vein, we analysed anti-LPS IgG levels in the blood, which reflect the host immune response to bacterial LPS. The levels were increased by the HFD compared with the NFD, and this increase was suppressed by LG2055 supplementation to the diet (Fig. 2(c)), implying the protective effect of LG2055 against the entry of bacterial LPS.

To identify whether the changes in gut permeability and anti-LPS IgG levels in the blood are associated with the adipose tissue inflammation, multiple correlation analysis between these parameters was performed. The M1:M2 ratio in adipose tissue was positively correlated with plasma FD-4 and anti-LPS IgG levels (Fig. 2(d) and (e)). In addition, there was a positive correlation between plasma FD-4 and anti-LPS IgG levels (Fig. 2(f)). These results suggest that the changes in gut permeability relate to adipose tissue inflammation.

### LG2055 prevents paracellular permeability increased by pro-inflammatory cytokines in Caco-2 cell monolayers

**Fig. 3.**
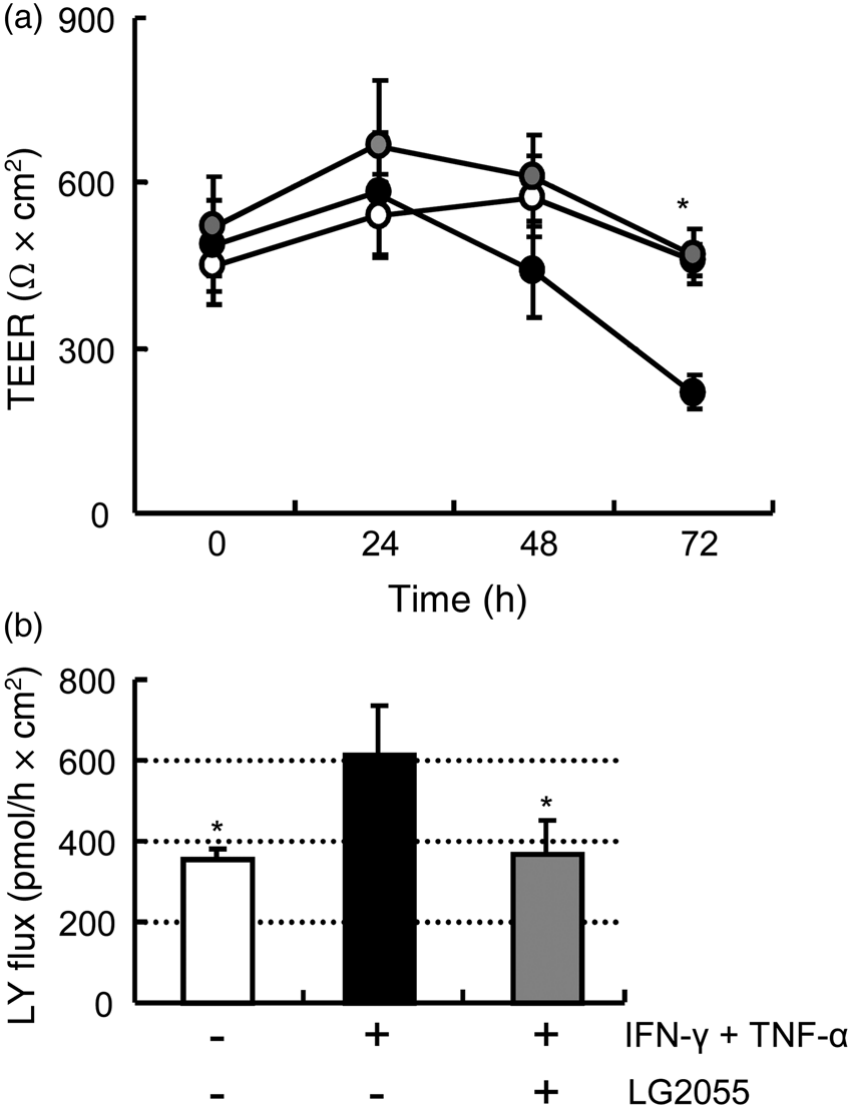
Effect of *Lactobacillus gasseri* SBT2055 (LG2055) on paracellular permeability of Caco-2 cell monolayers. Caco-2 cell monolayers were treated with 1 mg/ml LG2055 (apically), and 50 ng/ml interferon-γ (IFN-γ) and 10 ng/ml TNF-α (basally) for 72 h. (a) Trans-epithelial electric resistance (TEER) was measured 0, 24, 48 and 72 h after the treatment. –○–, Control; –●–, IFN-γ + TNF-α; –●◌–, IFN-γ + TNF-α + LG2055. (b) Rate of the Lucifer yellow (LY) flux across Caco-2 monolayers was measured 72 h after the treatment. Values are means, with standard deviations represented by vertical bars (*n* 3 in each group). Data were analysed by one-way ANOVA, followed by Dunnett's multiple-comparison test. * Mean value was significantly different from that of the IFN-γ + TNF-α group (*P* < 0·05).

As described above, LG2055 consumption prevented the increase in the intestinal permeability caused by the HFD in mice. We further examined the preventive effect of LG2055 on the barrier function using Caco-2 cell monolayers. Differentiated Caco-2 cells grown on a permeable support were basally treated with IFN-γ and TNF-α to induce impairment of the intestinal barrier for 72 h. We monitored TEER values every 24 h and paracellular permeability to LY across the Caco-2 monolayers after 72 h. When Caco-2 cell monolayers were treated with these cytokines, the TEER value was significantly reduced at 72 h (Fig. 3(a)) and the permeability to LY was significantly increased (Fig. 3(b)) compared with control without cytokines. The reduction in TEER and increase in LY permeability were both significantly ameliorated by the apical treatment with LG2055. These results suggest that LG2055 can directly affect epithelial cells to improve their barrier function.

## Discussion

A previous study showed that mice fed a HFD containing LG2055 cells had lower body and abdominal adipose tissue weights, and that consumption of LG2055 cells prevented the elevation of mRNA expression of pro-inflammatory markers *Ccl2, Ccr2*, and *Tnf* in adipose tissue^(^^15^^)^. Adipose tissue inflammation is initiated by the infiltration of macrophages and T cells into adipose tissue. On the other hand, recent studies suggested the disruption of intestinal barrier as a cause of inflammation in obesity^(^^4^^,^^21^^)^. These observations prompted us to investigate the effect of LG2055 on the immune cell population in adipose tissue and the barrier function of the intestine. Our present study demonstrated that mice fed a HFD (20 % (w/w)) had a remarkable gain in body weight and visceral fat mass compared with those fed a normal-fat (5 % (w/w)) diet (NFD), whereas a HFD containing LG2055 (HFD-LG) significantly suppressed the gain in body weight and mesenteric and perirenal/retroperitoneal adipose tissue weights. Thus, we initially confirmed that the present study successfully reached a state of diet-induced obesity and LG2055 improved the status, giving the rationale for the succeeding evaluation about whether LG2055 influences inflammatory status in adipose tissue and the intestinal barrier function.

The abundance of activated macrophages is a typical status of inflammatory adipose tissue and activated macrophages are classified into two groups: classically activated macrophages (M1) that produce pro-inflammatory cytokines such as TNF-α and IL-6, and alternatively activated macrophages (M2) that express anti-inflammatory markers such as arginase-1 and IL-10. While M2 macrophages are dominant in lean adipose tissue, the percentage of M1 macrophages increases as obesity progresses^(^^2^,^2^^)^. Thus, the balance between pro-inflammatory M1 and anti-inflammatory M2 macrophages plays an important role in the development of adipose tissue inflammation. The removal of CD11c-positive cells that include M1 macrophages remedies adipose tissue inflammation as well as insulin resistance in an obese mouse model^(^^2^,^3^^)^. Furthermore, macrophage-specific disruption of the PPAR-γ gene, which is required for the activation of M2 macrophages, aggravates adipose tissue inflammation and insulin resistance in diet-induced obese mice^(^^2^,^4^^)^, suggesting that M2 macrophages have protective effects against inflammation. In addition to macrophages, effector T cells are also involved in the progress of adipose tissue inflammation in obesity. A smaller number of CD4-positive T cells and a larger number of CD8-positive T cells were observed in the adipose tissue of obese mice compared with that of lean mice^(^^25^^,^^26^^)^. Nishimura *et al*. reported that immunological and genetic depletion of CD8-positive cells prevented adipose tissue inflammation and the development of systemic insulin resistance^(^^25^^)^. In the present study, HFD intake induced significant increases in M1 macrophages and the M1:M2 ratio, but a significant reduction in CD4-positive cells in adipose tissue. These results are consistent with observations in other studies on HFD-induced obese mice that showed an increase in M1 macrophages and a decrease in CD4-positive T cells in adipose tissue^(^^25^^,^^27^^,^^28^^)^. Such alterations generated by the HFD were modified by the consumption of LG2055 (HFD-LG), resulting in a significant increase in M2 macrophages and a significant decrease in the M1:M2 ratio in adipose tissue. Furthermore, the HFD-LG induced a significant reduction in CD8-positive cells in the tissue. Thus, LG2055 prevented the immune cell population in adipose tissue from being disposed towards inflammatory characteristics.

M2 macrophages are known to be induced by IL-4 and IL-13^(^^29^^)^ as well as by IL-10^(^^30^^)^. As LG2055 has been found to stimulate the IL-10 production of dendritic cells^(^^31^^)^, this might be partly responsible for the augmentation of M2-type macrophages by LG2055. It has also been reported that *L. paracasei* and *L. rhamnosus* prevented an increase in M1 macrophage markers (CD11c, MMP12) in adipose tissue during obesity^(^^32^^)^, and some lactobacilli ameliorated intestinal inflammation by polarising the macrophage population into M2 macrophages in the colon^(^^33^^,^^34^^)^. The present study also reports that *L. gasseri* is the first of these species shown to increase M2 macrophages in adipose tissue.

Disturbance of the intestinal barrier function is involved in adipose tissue inflammation in obesity^(^^4^^,^^35^^)^. An important function of the intestinal barrier is to prevent pro-inflammatory molecules, such as LPS of intestinal bacteria origin, from entering the systemic circulation. When the intestinal barrier is disrupted by HFD intake or genetic obesity, plasma LPS levels are elevated and play a critical role in developing systemic low-grade inflammation in obesity^(^^4^^,^^36^^,^^37^^)^. In our study, mice fed the HFD showed significantly higher levels of FITC–dextran (FD-4), which is poorly absorbed from the intestine, in plasma compared with mice fed NFD. The plasma FD-4 levels peaked at 1 h and then declined by 4 h, which is similar to other studies: for example, Cani *et al*. reported that FD-4-administered *Ob/Ob* mice showed that plasma FD-4 peaked at 1 h and then decreased^(^^20^^)^. Furthermore, an elevation in LPS-specific antibody levels was observed in the serum of mice fed the HFD, implying an increased entry of LPS across the gut barrier. By contrast, mice fed the HFD containing LG2055 (HFD-LG) showed significantly lower levels of FD-4 and anti-LPS antibodies in the blood compared with mice fed the HFD. These results imply that LG2055 prevented the intestinal barrier disruption and the entry of LPS across the gut epithelial barrier caused by HFD intake.

Intestinal barrier function is often evaluated using Caco-2 cell monolayers. *Bifidobacterium animalis* ssp. *lactis* 420, for instance, is able to enhance the tight junction barrier of Caco-2 monolayers^(^^38^^)^, which may be a mechanism for the prevention of the entry of LPS into the blood^(^^39^^)^. We evaluated whether LG2055 enhances the tight junction barrier using Caco-2 monolayers. The monolayers, basally treated with pro-inflammatory cytokines IFN-γ and TNF-α that are reported to be increased in plasma and intestine under obese or HFD-fed conditions^(^^35^^,^^40^^)^, increased their paracellular permeability, which was prevented by LG2055 cells apically added to the monolayers, suggesting that LG2055 cells enhanced the tight junction barrier. To further examine the improved tight junction barrier, we measured the expression levels of typical tight junction proteins (ZO-1, occludin and claudin-4) in Caco-2 cells as well as in the intestinal epithelial cells prepared from mice fed LG2055 by Western blotting. However, no effects of LG2055 on the tight junction proteins were observed in either sample (data not shown). The integrity of the tight junction is regulated by not only the expression levels but also the distribution of tight junction proteins (localisations of tight junction proteins to the plasma membranes or to the cytosol)^(^^41^^)^. For example, *L. plantarum*, which belongs to the same genus as LG2055, attenuated the dislocation of ZO-1 and occludin and prevented an increase in the paracellular permeability of Caco-2 monolayers treated with phorbol ester^(^^42^^)^. We also consider it important to further examine whether LG2055 enhances the barrier function through improving the distribution of the tight junction proteins.

In conclusion, LG2055 prevented body-weight gain, visceral fat accumulation and polarisation of macrophages into an inflammatory subtype in adipose tissue in mice fed a HFD. Consumption of LG2055 also prevented high-fat-induced increases in intestinal permeability and anti-LPS antibody levels in the blood, implying an inhibited entry of LPS across the gut epithelial barrier. Addition of LG2055 cells to Caco-2 cell monolayers inhibited paracellular permeability elevated by inflammatory cytokines. Thus, the enhancement of intestinal barrier function may reduce the entry of inflammatory substances such as LPS from the intestine into the circulation, contributing to the inhibition of adipose tissue inflammation and obesity.
